# Impact of Laparoscopic Sleeve Gastrectomy on Fatigue in Obese Patients

**DOI:** 10.3390/jcm13164746

**Published:** 2024-08-13

**Authors:** Ilhan Tasdoven, Hakan Balbaloglu

**Affiliations:** Department of General Surgery, School of Medicine, Bülent Ecevit University, Zonguldak 67000, Turkey; hakanbalbaloglu@yahoo.com

**Keywords:** Fatigue Assessment Scale, sleeve gastrectomy, obesity, fatigue, quality of life

## Abstract

**Background:** Fatigue with obesity negatively affects the motivation to lose weight and causes failure of treatment. So, obesity and fatigue are two related entities that worsen each other in a vicious circle. This study aimed to examine the changes in fatigue levels in obese patients undergoing laparoscopic sleeve gastrectomy (LSG). **Methods:** Preoperative and postoperative BMI, fat percentage, and obesity degree were determined based on Tanita test results, and the rates of change were calculated. The Fatigue Assessment Scale (FAS) was used to assess the impact of obesity on mental and physical fatigue. **Results:** Six months after LSG, there was a 29.7% decrease in Body Mass Index (BMI) across all patients (45.0 to 31.4, *p* < 0.001). Significant reductions in the percentage of body fat, obesity grade, and HgA1C were observed in both women and men and overall (*p* < 0.001 for all comparisons). Scores on the Fatigue Assessment Scale (FAS) for total, mental, and physical scales decreased significantly for women, men, and all participants (*p* < 0.001 for all comparisons). There was a weak but significant positive correlation between the percentage change in FAS total and FAS physical scale scores and the change in BMI% (*p* = 0.015 and 0.004; r = 0.089, 0.106, respectively). Change in obesity grade and FAS physical subscale scores negatively correlated (*p* = 0.029, r = 0.080). **Conclusions:** LSG not only leads to significant weight reduction but also improves fatigue levels in patients with obesity.

## 1. Introduction/Purpose

Obesity, along with its associated comorbidities and complications such as hypertension, dyslipidemia, and obstructive sleep apnea syndrome is rapidly increasing worldwide. It is estimated that 39% of the population aged 18 and over worldwide is overweight and 14% is obese, and, in our country, the obesity rate is approximately 20% according to 2019 data [1]. These conditions have the potential to affect up to one billion people, leading to increased morbidity and mortality, reduced quality of life, and rising healthcare costs [2,3,4]. Pharmacological and surgical interventions for obesity can significantly reduce both the medical and societal costs associated with healthcare. In obesity treatment, bariatric surgery (also known as metabolic and bariatric surgery (MBS)) is considered when medical treatment, lifestyle changes, and conservative treatments are ineffective. It is also considered for individuals with a BMI greater than 40 kg/m^2^ or a BMI between 35 and 40 kg/m^2^ with obesity-related complications such as hypertension, dyslipidemia, and obstructive sleep apnea [4,5]. Current meta-analyses support that MBS is an effective option for the treatment of obesity and related complications [6,7,8].

The International Federation for the Surgery of Obesity and Metabolic Disorders (IFSO) and the American Society for Metabolic and Bariatric Surgery (ASMBS) have reported that obesity surgery, the most commonly performed bariatric surgery worldwide, is effective for weight loss and diabetes regression even within one year [5,9]. Recent reports indicate that the most commonly performed surgical procedures worldwide are sleeve gastrectomy (SG) and Roux-en-Y Gastric Bypass (RYGB) [5,9]. Laparoscopic sleeve gastrectomy (SG) has become increasingly prominent in metabolic and bariatric surgery (MBS) methods over the years thanks to advancements in medical technology [10]. Systematic meta-analyses recommend sleeve gastrectomy (SG) worldwide, particularly for high-risk patients, pediatric patients, and adults over 65 years of age. However, it is not recommended for patients with type 2 diabetes, gastroesophageal reflux disease, or non-alcoholic steatohepatitis [3,5,9].

Obesity and the metabolic disease that it causes damage almost every organ system. These include the cardiovascular system (coronary artery disease, hypertension, dyslipidemia, heart failure, and cerebrovascular events), respiratory system (obstructive sleep apnea syndrome (OSAS) and asthma), gastrointestinal syndrome (gastroesophageal reflux disease (GERD), cholelithiasis, cholecystitis, and pancreatitis), endocrine system (insulin resistance and type 2 diabetes). On the other hand, obesity negatively affects the reproductive system (polycystic ovary syndrome), liver diseases (fatty liver and NASH), kidneys (nephrolithiasis and chronic kidney disease), musculoskeletal system (osteoarthritis and muscle weakness), and psychosocial and behavioral health (depression and suicide). It also increases the risk of some organ cancers [5,11,12,13,14,15,16]. 

Severe obesity causes a slow activation of the innate immune system through increased circulating plasma concentrations of pro-inflammatory cytokines. The release of inflammatory chemokines (necrosis factor (TNF) alpha, interleukin (IL)-1 and IL-6, plasminogen activator inhibitor-1 (PAI-1), C-reactive protein (CRP), and leptin) stimulates T cell activation and causes the infiltration of macrophages [17,18]. In contrast, anti-inflammatory modulators such as adiponectin decrease with increasing adipose tissue, and obesity progresses as a low-grade chronic inflammatory disease [19]. Also, the limitation of physical and social activities caused by being overweight and the negative interaction in the psychosocial or musculoskeletal system are associated with low energy levels and chronic fatigue. Obesity and its related comorbidities can lead to reduced energy levels and fatigue, which in turn restrict physical activity. This can negatively affect the motivation to lose weight and exacerbate related health conditions. Fatigue levels are an important indicator of the quality of life in chronic diseases and are widely studied. Therefore, monitoring fatigue closely is recommended in obesity management [20,21,22]. A reduction in fatigue levels helps obese patients to achieve weight loss and prevent losses in the musculoskeletal system by increasing compliance with exercise programs both before and after MBS [23,24]. Uncontrolled fatigue levels not only make the treatment of obesity and related diseases difficult but also cause the progression of these diseases both physically and mentally. Fatigue, which affects mental and physical functions, stands out as a complicating factor in obese patients, as in other chronic diseases. Still, fatigue has recently begun to enter the field of interest of researchers, and there is a lack of data on obesity and fatigue levels.

The aim of this study was to examine changes in fatigue levels from the preoperative to the postoperative period and to explore their relationship with weight loss outcomes in patients undergoing LSG.

## 2. Patients and Methods

Study design: This study was designed prospectively. 

Setting: This study was conducted among patients who underwent laparoscopic sleeve gastrectomy (LSG) for obesity at the Department of General Surgery, Faculty of Medicine, of XXX University between June 2022 and March 2024. Patients were included in this study after evaluations by physicians from the departments of Endocrinology and Metabolism, Chest Diseases, Cardiology, Gastroenterology, General Surgery, Psychiatry, and Anesthesia at the university’s obesity center.

Eligibility criteria: Patients with a diagnosis of Class 1 or 2 obesity, according to the current guidelines of the Society of American Gastroenterological Surgeons, IFSO, and ASMBS, were selected for this study. The inclusion criteria were as follows: (a) a BMI greater than 40 kg/m^2^ or (b) a BMI greater than 35 kg/m^2^ with at least one comorbid condition (such as type 2 diabetes, hypertension, dyslipidemia, or obstructive sleep apnea), as well as an age between 18 and 60 years and the inability to lose weight through medical treatment for at least 2 years [25]. 

Among the patients who underwent MBS, only those who underwent LSG and received regular information and postoperative follow-up were included in the study. Revision MBS patients, patients with a BMI > 60 kg/m^2^, patients with uncontrolled organic or psychiatric diseases (body dysmorphic disorder or hypomania/depression), patients with alcohol dependence, patients with an active peptic or duodenal ulcer, patients with a refractory GERD with a large hiatal hernia, patients with a history of previous major abdominal surgery, and patients with a previous diagnosis of abdominal organ malignancy were excluded. Participants diagnosed with other chronic diseases associated with chronic fatigue and also unrelated to obesity (rheumatic, hematological, etc.) were not included in this study.

We recorded the age and gender of the patients. BMI, percentage of body fat, and degree of obesity were measured both preoperatively (preop) and at the sixth postoperative (postop) month. These measurements were based on the preoperative gastroscopy and biopsy results for eligible patients, as well as on preoperative and postoperative Tanita test results [26]. To assess the impact of the operation on mental and physical fatigue, we used the Fatigue Assessment Scale (FAS). Data were collected through face-to-face interviews both before and after surgery. The FAS scale comprises mental and physical subscales, totaling 10 items. The FAS is a validated inventory that can also reflect emotional fatigue levels. Each item offers 5 Likert-type response options, ranging from ‘never’ to ‘always’ [27].

Statistical analysis: SPSS 27.0 (IBM Corporation, Armonk, New York, NY, USA) and PAST 3 (https://www.uv.es/~pardomv/pe/2001_1/past/pastprog/past.pdf, accessed on 4 August 2024) programs were used to analyze the variables. The conformity of univariate data to normal distribution was evaluated by the Kolmogorov–Smirnov test. The Mardia test (Dornik and Hansen omnibus) was used to assess the conformity of multivariate data to normal distribution. The Box-M test was used for variance homogeneity. We used the Mann –Whitney U test with Monte Carlo results to compare two independent groups based on quantitative variables. To compare two repeated measurements of dependent quantitative variables, we used the Wilcoxon signed-rank test with Monte Carlo simulation results. The partial correlation test was used to examine correlations between variables after controlling for gender effects. Quantitative variables are presented in tables as medians (1st quartile/3rd quartile). All analyses were conducted at a 95% confidence level, and a *p*-value of less than 0.05 was considered statistically significant.

Ethic Committee Name: The Institutional Ethics Board of University, the School of Medicine, reviewed and approved this study’s protocol. Approval Code: 2022/10 (approval date: 25 May 2022).

## 3. Results

**Figure 1:**
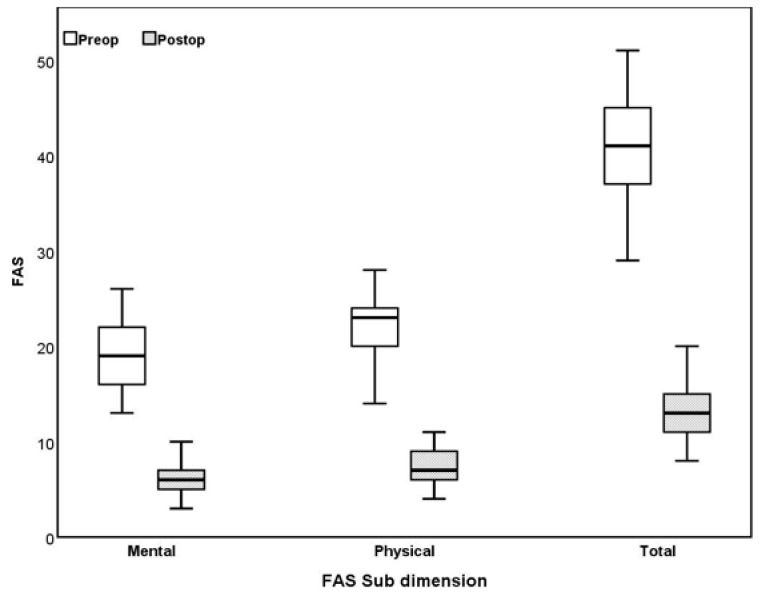
Box and line chart for Fatigue Assessment Scale (FAS). FAS subscale scores and total scores were significantly decreased after surgery.

This study comprised 267 women (71.8%) and 105 men (28.2%). The average age of the patients was 37.9 years, with a range from 17 to 67 years (mean ± SD: 37.9 ± 10.7). There was no significant difference in age between men and women (*p* = 0.354). Six months after MBS, patients experienced a 29.7% reduction in BMI, dropping from an average of 45.0 to 31.4 (BMI%, *p* < 0.001). The percentage of body fat also significantly decreased in all groups: women, men, and overall (*p* < 0.001 for each group). The degree of obesity showed substantial improvement, decreasing by 61.8% in women, 60.7% in men, and 61.4% overall (*p* < 0.001 for each group). HbA1C levels decreased significantly by 19.7%, from an average of 5.8 to 5.3, among all patients (*p* < 0.001). FAS mental scale scores showed a significant decrease in women, men, and in overall participants by 65.4%, 66.7%, and 66.7%, respectively (*p* < 0.001 for each group). Similarly, FAS physical subscale scores also significantly decreased by 67.5%, 68.1%, and 67.5% in women, men, and overall participants, respectively (*p* < 0.001 for each group, Figure 1). The distribution and statistical analysis of the findings for women, men, and overall participants are presented in Table 1. We found a weak positive correlation between the percentage change in FAS total scores and FAS physical subscale scores and BMI% change. However, there was no correlation between changes in FAS mental scale scores and BMI (*p* = 0.015, 0.004, and 0.207; r = 0.089, 0.106, and 0.046, respectively). Additionally, a weak but significant negative correlation was observed between the percentage decrease in the degree of obesity and the percentage change in FAS physical subscale scores (*p* = 0.029, r = 0.080, Table 2).

## 4. Discussion

Available studies on LSG indicate several advantages, including optimal weight loss, regression of comorbid diseases, ease of technique, shorter operation and hospitalization times, reduced use of foreign objects, and limited immediate caloric intake. In both short-term and mid-term follow-ups, LSG has shown lower operative morbidity compared to gastric bypass techniques. GERD, failure to achieve adequate weight loss, weight regain, and revision after SG are the main causes of MBS. Additionally, SG is recommended as a first-line or stand-alone surgical technique for high-risk patients, including those of advanced age (>65 years), who may require multistage MBS techniques [9,25,28]. In this study, FAS total scores, as well as both the mental and physical subscale scores, decreased significantly among women, men, and all participants. However, we found only a weak and significant correlation between the percentage change in FAS total scores and FAS physical subscale scores and BMI% change. Additionally, there was a weak inverse relationship between the percentage decrease in the degree of obesity and the change in FAS physical subscale scores. These findings suggest that while fatigue levels improve with weight loss, rapid weight loss post-surgery may actually increase fatigue levels. Additionally, the presence of comorbid diseases, changes in muscle strength, metabolic factors, and biochemical changes may influence fatigue levels.

Obesity and comorbid diseases are closely linked to low energy and fatigue levels, similar to other chronic conditions. Limited activity can exacerbate obesity and its associated health issues [20,21]. Feeling tired or energized is an important indicator of health and quality of life, reflecting both physical and mental well-being. This aspect is widely studied in research [22,29]. It is a known fact that patients who are candidates for bariatric surgery often experience persistent fatigue and low energy levels [30,31]. Consistent with the findings of this study, previous research has shown that energy and fatigue levels improve in patients after MBS. Specifically, existing studies indicate that fatigue levels decrease as the total weight loss (TWL) percentage increases, often due to reduced physical activity restrictions following surgery [29,32,33,34]. Improvement in energy or fatigue after MBS could be related to weight loss and/or increased engagement in daily health behaviors such as physical activity [35]. Also, MBS induces the production of pro-inflammatory cytokines, such as CRP, IL-6, IL-10, and IL-18, postoperatively [36]. In previous studies, patients’ energy or fatigue levels have often been examined as indicators of physical activity, or these levels have been included within subgroups of broader quality-of-life scales. Soroceanu et al. found that patients undergoing MBS scored lower on the energy/fatigue subscale of the 36-Item Short Form Survey (SF-36), which also includes eight other subcategories such as broader physical functions and limitations in social roles. These scores varied depending on the type of surgery. In this study, it was suggested that different subscales in quality-of-life questionnaires should be evaluated separately [22]. Impellizzeri et al. adapted the Fatigue Severity Scale (FSS) to patients with obesity and recommended the use of fatigue level in clinics and studies [30]. The Multidimensional Fatigue Symptom Inventory (MFI) is another fatigue scale that has been adapted for use in both pediatric and adult obese patients. In contrast, the use of the FAS scale among obese patients is less common in the literature. The FAS scale includes subscales for evaluating mental and physical fatigue. Like the FSS scale, it consists of 10 items, but the response options differ [30,37]. Gletsu-Miller et al. [38] found no significant improvement in fatigue levels during the first postoperative month using the MFI scale in patients who underwent RYGB. However, they observed significant improvements in both physical and mental fatigue by the sixth postoperative month. In our study, an improvement in mental fatigue levels, particularly by the sixth month, was associated with a decrease in the abdominal visceral fat ratio. Among the other factors we examined—total fat, subcutaneous fat, insulin resistance, and interleukin-6 (IL-6)—none had an impact on fatigue levels. Similarly, changes in fat percentage did not correlate with improvements in HbA1C levels [38]. 

Schumacher et al. [20] conducted a prospective study where they monitored moderate-to-vigorous intensity physical activity (MVPA) using both ecological momentary assessment (EMA) scales and accelerometry. They tracked these scores from before metabolic and bariatric surgery (MBS) up to one year after the surgery. The study found that energy levels increased in the first month following surgery but remained relatively stable between the third and twelfth months. Additionally, attention and fatigue levels showed no significant change compared to the preoperative period. There was no notable correlation between the total weight loss (TWL) ratio and these variables. However, a significant correlation was observed between changes in MVPA and levels of energy and attention. In a prospective cohort study investigating physical activity and dietary habits following MBS, Bond et al. found that moderate-to-vigorous physical activity (MVPA) scores increased for patients in both the SG and RYGB groups. However, daily sedentary time remained unchanged. Interestingly, the study revealed that post-surgery energy intake restriction affected MVPA scores specifically in the RYGB group [33].

Studies in the field of quality of life have previously highlighted the importance of assessing fatigue levels in obese patients and evaluating them in the context of comorbid diseases [20,21,30]. Fatigue not only diminishes the quality of life for overweight patients, but it also exacerbates obesity itself. It hinders obesity treatment by limiting physical activity and further worsening the condition. Reduced physical activity due to fatigue can make it challenging for patients to manage their weight. This creates a vicious cycle, making weight loss efforts more difficult and leading to a self-perpetuating situation [20,21]. The assessment of fatigue levels in obese patients and their impact on outcomes following MBS have become increasingly important topics in current obesity research [20,21,22,30]. Fatigue levels in obese patients cannot be solely attributed to total weight loss. For instance, obstructive sleep apnea syndrome (OSAS), commonly associated with obesity, correlates with higher Fatigue Severity Scale (FSS) scores, particularly in men with a higher apnea–hypopnea index [39]. Muscle weakness, joint diseases, and depression can contribute to overall fatigue by restricting physical and social activities. Furthermore, variations in fatigue scales and components of broader quality of life measures can complicate interpretation. Nonetheless, fatigue levels tend to decrease in the medium term following metabolic and bariatric surgery (MBS). It is highly recommended that fatigue levels be assessed in obese patients and in studies evaluating the effectiveness of MBS, along with monitoring other comorbid conditions. 

Strengths and limitations: Excluding patients with other comorbid diseases such as OSAS can decrease the homogeneity of the sample. Additionally, not investigating other biochemical factors related to fatigue levels could be a limitation. Including a larger number of patients in the study would naturally enhance the generalizability of the results. In particular, potential bilateral bias in patient selection may have made it difficult to detect a clear and high correlation between total weight loss and energy levels, as expected. On the other hand, since fatigue is an abstract issue that may have many underlying layers in terms of mental and physical aspects, its relationship with total weight loss may be weaker than we thought.

## 5. Conclusions

MBS, including LSG, has been shown to improve various levels of fatigue. Incorporating fatigue level assessments into clinical trials could aid in obesity management. However, the underlying mechanism for this improvement remains unclear. It is essential to conduct studies in the future with multifactorial analyses that consider obesity-related diseases, metabolic conditions, biochemical factors that could influence fatigue levels, the long-term effects of MBS on fatigue, and different subgroups of patients.

## Figures and Tables

**Table 1:** Distribution of patients’ Fatigue Assessment Scale scores, HgA1C values, and weight loss parameters and their changes according to preoperative and postoperative periods by gender.

	Total (*n* = 372)	Female (*n* = 267)	Male (*n* = 105)	*p*
	Median (q1–q3)	Median (q1–q3)	Median (q1–q3)	
Age	37.0 (30.0–44.0)	36.0 (29.0–45.0)	38.0 (33.0–43.0)	0.354 ^u^
BMI				
Preop	45.0 (42.0–47.2)	45.0 (42.0–47.1)	45.5 (42.0–48.3)	0.317 ^u^
Postop	31.4 (28.7–34.1)	31.4 (28.7–33.5)	31.6 (28.6–35.2)	0.498 ^u^
% Change	29.7 (20.4–37.7)	30.0 (20.4–37.5)	29.2 (20.4–39.9)	0.647 ^u^
*p*-value for preop vs. postop	<0.001 ^W^	<0.001 ^W^	<0.001 ^W^	
Fat %				
Preop	41.8 (36.6–45.6)	42.3 (36.8–45.6)	40.1 (35.8–45.0)	0.056 ^u^
Postop	27.4 (24.8–30.9)	27.4 (25.0–31.0)	26.9 (24.6–30.5)	0.392 ^u^
% Change	33.4 (24.3–40.2)	33.6 (24.4–40.2)	33.4 (24.3–39.9)	0.482 ^u^
*p*-value for preop vs. postop	<0.001 ^W^	<0.001 ^W^	<0.001 ^W^	
Obesity degree				
Preop	79.4 (69.8–98.5)	80.2 (70.0–96.8)	78.5 (69.8–98.5)	0.732 ^u^
Postop	31.5 (27.4–34.8)	31.7 (27.7–34.8)	30.1 (26.8–34.5)	0.362 ^u^
% Change	61.4 (52.4–69.2)	61.8 (51.8–68.9)	60.7 (53.2–69.8)	0.827 ^u^
*p*-value for preop vs. postop	<0.001 ^W^	<0.001 ^W^	<0.001 ^W^	
HbA1c				
Preop	5.8 (5.4–6.8)	5.8 (5.3–6.8)	5.9 (5.6–7.0)	0.161 ^u^
Postop	5.3 (4.6–5.9)	5.3 (4.6–5.9)	5.3 (4.2–5.8)	0.507 ^u^
% Change	19.7 (8.9–35.8)	19.4 (9.3–34.5)	23.9 (8.8–37.9)	0.292 ^u^
*p*-value for preop vs. postop	<0.001 ^W^	<0.001 ^W^	<0.001 ^W^	
FAS mental				
Preop	19.0 (16.0–22.0)	19.0 (16.0–22.0)	19.0 (16.0–22.0)	0.387 ^u^
Postop	6.0 (5.0–7.0)	6.0 (5.0–7.0)	6.0 (5.0–7.0)	0.948 ^u^
% Change	70.0 (62.5–75.0)	70.6 (62.5–75.0)	69.6 (61.9–75.0)	0.582 ^u^
*p*-value for preop vs. postop	<0.001 ^W^	<0.001 ^W^	<0.001 ^W^	
FAS physical				
Preop	23.0 (20.0–24.0)	23.0 (20.0–24.0)	22.0 (20.0–24.0)	0.927 ^u^
Postop	7.0 (6.0–9.0)	7.0 (6.0–9.0)	8.0 (6.0–9.0)	0.781 ^u^
% Change	66.7 (60.9–72.7)	65.4 (61.1–72.0)	66.7 (60.0–73.1)	0.925 ^u^
*p*-value for preop vs. postop	<0.001 ^W^	<0.001 ^W^	<0.001 ^W^	
FAS total				
Preop	41.0 (37.0–45.0)	41.0 (37.0–45.0)	41.0 (37.0–45.0)	0.551 ^u^
Postop	13.0 (11.0–15.0)	13.0 (11.0–15.0)	13.0 (12.0–15.0)	0.949 ^u^
% Change	67.5 (63.2–72.5)	67.5 (63.6–72.5)	68.1 (62.5–72.5)	0.752 ^u^
*p*-value for preop vs. postop	<0.001 ^W^	<0.001 ^W^	<0.001 ^W^	

^u^ Mann–Whitney U Test (Monte Carlo), ^W^ Wilcoxon signed-rank test (Monte Carlo), Q1: 1st quartile, Q3: 3rd quartile.

**Table 2:** Correlation table showing the changes in FAS scale scores, HgA1C values, and weight loss parameters of the patients.

	Fatigue Assessment Scale % Change		
	Mental	Physical	Total
Age			
r	−0.030	−0.007	−0.020
*p*	0.410	0.848	0.584
BMI % change			
r	0.046	0.106	0.089
*p*	0.207	0.004	0.015
Fat % change			
r	0.041	0.067	0.066
*p*	0.260	0.068	0.071
% Degree of obesity change			
r	−0.013	−0.080	−0.058
*p*	0.727	0.029	0.117
HbA1c % change			
r	−0.012	−0.035	−0.047
*p*	0.754	0.339	0.200

Partial correlation test; gender effect is controlled. r: correlation coefficient.

## Data Availability

Data available on request due to restrictions, e.g., privacy or ethical.
